# TNF-α-induced miR-450a mediates TMEM182 expression to promote oral squamous cell carcinoma motility

**DOI:** 10.1371/journal.pone.0213463

**Published:** 2019-03-20

**Authors:** En-Wei Hsing, Shine-Gwo Shiah, Hsuan-Yu Peng, Ya-Wen Chen, Chih-Pin Chuu, Jenn-Ren Hsiao, Ping-Chiang Lyu, Jang-Yang Chang

**Affiliations:** 1 National Institute of Cancer Research, National Health Research Institutes, Miaoli, Taiwan; 2 Structural Biology Program, Institute of Bioinformatics and Structural Biology, National Tsing Hua University, Hsinchu, Taiwan; 3 Institute of Cellular and System Medicine, National Health Research Institutes, Miaoli, Taiwan; 4 Department of Otolaryngology, Head and Neck Collaborative Oncology Group, National Cheng Kung University Hospital, College of Medicine, National Cheng Kung University, Tainan, Taiwan; 5 Division of Hematology and Oncology, Department of Internal Medicine, National Cheng Kung University Hospital, College of Medicine, National Cheng Kung University, Tainan, Taiwan; University of South Alabama Mitchell Cancer Institute, UNITED STATES

## Abstract

Distant metastasis leads oral cancer patients into a poor survival rate and a high recurrence stage. During tumor progression, dysregulated microRNAs (miRNAs) have been reported to involve tumor initiation and modulate oral cancer malignancy. MiR-450a was significantly upregulated in oral squamous cell carcinoma (OSCC) patients without functional reports. This study was attempted to uncover the molecular mechanism of novel miR-450a in OSCC. Mir-450a expression was examined by quantitative RT-PCR, both in OSCC cell lines and patients. Specific target of miR-450a was determined by software prediction, luciferase reporter assay, and correlation with target protein expression. The functions of miR-450a and TMEM182 were accessed by adhesion and transwell invasion analyses. Determination of the expression and cellular localization of TMEM182 was examined by RT-PCR and by immunofluorescence staining. The signaling pathways involved in regulation of miR-450a were investigated using the kinase inhibitors. Overexpression of miR-450a in OSCC cells impaired cell adhesion ability and induced invasiveness, which demonstrated the functional role of miR-450a as an onco-miRNA. Interestingly, tumor necrosis factor alpha (TNF-α)-mediated expression of TMEM182 was regulated by miR-450a induction. MiR-450a-reduced cellular adhesion was abolished by TMEM182 restoration. Furthermore, the oncogenic activity of TNF-α/miR-450a/TMEM182 axis was primarily through activating extracellular signal–regulated kinase 1/2 (ERK1/2) signaling pathway. ERK1/2 inhibitor prevented the TNF-α-induced miR-450a expression and enhanced adhesion ability. Our data suggested that TNF-α-induced ERK1/2-dependent miR-450a against TMEM182 expression exerted a great influence on increasing OSCC motility. Overall, our results provide novel molecular insights into how TNF-α contributes to oral carcinogenesis through miR-450a that targets TMEM182.

## Introduction

Oral squamous cell carcinoma (OSCC) is the most lethal type in head and neck squamous cell carcinoma (HNSCC) in the world. Over the past decades, the incidence rate of OSCC has increased among younger generations [1–3]. In spite of considerable advances in surgery, radiotherapy and chemotherapy, the 5-year survival rate for OSCC has not improved markedly because patients still frequently arise loco-regional recurrence and lymph node metastasis [4–6]. Hence, finding new biomarker(s) and therapeutic molecule(s) is urgent. Recently, a growing evidence indicates that microRNAs (miRNAs) contribute to the initiation and development of oral cancer [7–9]. Therefore, exploring unique miRNAs and related molecular pathways underlying OSCC aggressive will provide advantages to improve therapeutic efficacy, as well as to design more effective treatment strategies.

Previously, we established a dysregulated signature of eighty-four miRNAs from OSCC clinical samples using a miRNA microarray [10]. From this analysis, we found that miR-450a was significantly overexpressed in tumor tissues than that in corresponding adjacent normal tissues. MiR-450a is an intragenenic miRNA clustered with miR-542-5p, miR-542-3p, miR-503, miR-450b-5p, miR-450b-3p, and miR-424 on chromosomal location Xq26.3. High expression level of miR-450a performs as a potential oncogene in laryngeal squamous cell carcinoma and breast cancer [11, 12]. Up-regulated miR-450a is found in mesenchymal part of epithelial-to-mesenchymal transition (EMT)-activation in human endometrial carcinosarcoma [13]. Contrarily, downregulation of miR-450a is required in hepatocellular carcinoma carcinogenesis [14]. Although miR-450a was reported to be dysregulated in different cancer types, its functions and underlying mechanisms have not been elucidated, especially in oral cancer.

Transmembrane proteins (TMEMs) are a group of novel proteins, which have key roles in cell differentiation and tumorigenesis in many cancers, such as pancreatic cancer, prostate cancer, ovarian cancer, and renal cell carcinoma [15–19]. However, the role of TMEM182 in oral cancer is unknown. In this study, we demonstrated that miR-450a may function as an oncogene by directly targeting 3’-untranslated region (UTR) of TMEM182 and reduce its expression in OSCC. Downregulation of TMEM182 or overexpression of miR-450a has the same effect on reducing cellular adhesion of OSCC cells. In addition, we also found that miR-450a expression was increased by TNF-α through ERK1/2-dependent pathway, more than via NF-κB. Taken together, these findings may provide understanding into oral carcinogenesis and suggest new therapeutic opportunities in this cancer.

## Materials and methods

### Human samples

The study protocol was approved by the Research Ethics Committee of National Health Research Institutes (EC1040409-E) and Institutional Human Experiment and Ethic Committee of National Cheng Kung University Hospital (HR-97-100) for the use of clinical materials for research purpose. Thirty-five paired primary OSCC and their adjacent non-tumorous epithelial samples were obtained from patients with curative surgery at the National Cheng Kung University Hospital (Tainan, Taiwan) from 1999 to 2010. All human tissues were snap-frozen in liquid nitrogen. Total RNA was extracted by miRNeasy Mini Kit (Qiagen, #217004) followed by instruction manual. The patient’s backgrounds and clinical parameters were summarized in S1 Table. The prognostic value of miR-450a among The Cancer Genome Atlas (TCGA) HNSCC cohort was analyzed through SurvMicro database (http://bioinformatica.mty.itesm.mx:8080/Biomatec/Survmicro.jsp) and was uploaded to GEO (Access number GSE36682). Forty paired of OSCC patients cDNA microarrays analysis were performed according to our previous study deposited in GEO (Accession number GSE37991 and GSE45238) [10].

### Cell culture

Cultured conditions for all human OSCC cell lines were summarized as previously described [15]. Human oral keratinocytes (HOK) were purchased from ScienCell (Carlsbad, CA, USA) and cultured according to the manufacturer’s instructions. All cells were maintained at 37°Clin a 5% CO_2_ atmosphere properly.

### Cytokine and chemical inhibitor treatment

Cells were incubated at low-serum conditional medium for 24 h, before TNF-α (10ng/ml) addition as indicated times. For intrinsic pathway analysis, cells were incubated with each of the following inhibitors: 1 μM for human dysplasia oral keratinocyte (DOK) and 10 μM for human tongue cancer cells (SAS) of NFκB inhibitor (Calbiochem, 481406), 30 μM U0126 (ERK inhibitor)(Cell Signaling, 9903), and 30 μM SB203580 (p38 inhibitor)(Cell Signaling, 5633).

### RNA extraction and reverse-transcription PCR (RT-PCR)

Total RNA was extracted from OSCC cell lines using TRIzol reagent (Life Technologies, Gaithersburg, MD) according to the manufacturer’s instructions. RNA concentration and purification were checked by NanoDrop ND-1000 spectrophotometer. First-strand cDNAs were synthesized by NxGen^™^M-MuLV reverse transcriptase with oligo dT_12-18_ primer (Invitrogen, Carlsbad, CA). Gene expression analyses were assayed on a Biometra T3000 thermocycler (Biometra GmbH, Germany) as following conditions: 95°C for 5 min, followed by 35–40 cycles of amplification (95°C for 30s, 60°C for 30s, and 72°C for 30s), and 72°C for 10 min. GAPDH was used as a loading control. PCR products were subjected to electrophoresis on 2% agarose gel and visualized on UVP GDS-8000 Bioimaging System (UVP, CA, USA) with 0.01% of SYBRSafe (Invitrogen, Carlsbad, CA, USA) inner staining. Primer sequences are listed in S2 Table.

### Quantitative real-time PCR (qPCR)

Mature miR-450a and RNU44 internal control levels were analyzed by miRNA-specific stem-loop primers and TaqMan Universal PCR Master Mix (Applied Biosystems) on an Applied Biosystems StepOne Plus real-time PCR system. Fold changes were calculated by using2^-ΔΔCt^ method using control and reference normalized. Primer sequences are listed in S2 Table.

### Plasmids

A wild type 3′-UTR of TMEM182 containing the miR-450a binding sites (3'UTR-WT) and truncated 3′-UTR fragment with deleted miR-450a binding sites (3'UTR-DEL) were constructed into the XhoI/XbaI sites of pmiRGLO firefly luciferase-expressing vector (Promega, WI, USA). For gene knockdown experiments, the shRNA clones of TMEM182 (sh182 #1 & #2) and empty vector pLKO_TRC (shCTRL) were obtained from the National RNAi Core Facility (Academia Sinica, Taiwan). Human TMEM182 cDNA was sub-cloned into empty vector pCDH-CMV-GFP puro+ (vehicle) (System Biosciences) at EcoRI/BamHI sites and termed as TMEM182-flag. Human TMEM182 cDNA was sub-cloned into empty vector pEGFPN1 (BD Biosciences Clontech’s) (vehicle) at XhoI/BamHI sites, termed as TMEM182-GFP. The sequence data were compared against the National Center for Biotechnology Information (NCBI) database using BLAST and miRBase (http://www.mirbase.org/). Kyte-Doolittle hydrophobicity analysis was accessed to predict TMEM182 transmembrane portions using ExPASY (https://web.expasy.org/protscale/). List primer sequences in the following S2 Table.

### Protein extraction and western blot

Cell lysates were prepared as previously reported.[20] Equal amounts of protein lysates were separated by 10~12% SDS polyacrylamide gels and transferred to poly-vinylidene fluoride (PVDF) membrane (Pall Life Sciences, Glen Cove, NY). Immunoblotting was performed with specific antibodies against TMEM182 (ab177360; Abcam). α-tubulin (sc-23950; Santa Cruz) and GAPDH (GeneTex, GTX100118) were used as internal controls. Signals from HRP-conjugated secondary antibodies were visualized by enhanced chemiluminescence (ECL) detection system (PerkinElmer, Waltham, MA) and chemiluminescence was exposed onto Kodak X-Omat film (Kodak, Chalon/Paris, France).

### Microscopic examination and immunofluorescence staining

Optic microscopy with 40 magnification objective lens was used for cell morphology and adhesion assays. Images were analyzed with ImageJ software. Scale bars were indicated in panels. In immunofluorescence staining, SAS cells were transfected with TMEM182-GFP or empty vector plasmids for 36 h, following by gently fixed with 4% paraformaldehyde and permeabilized with phosphate buffered saline (PBS) containing 0.1% Triton X-100. Slides were stained with mouse polyclonal antibody against E-cadherin antibody (1:1000, Cell signaling 3195) at 4°C overnight. 2^nd^ donkey polyclonal antibody conjugated with TexasRed (Santa Cruz, 1:1000) were used for 1 h at room temperature and mounted using mounting solution (ImmunoTech). DAPI (Roche) was used as counterstain for 1 h. Slides were examined by Leica TCS SP5 and analyzed with ImageJ software.

### Transfection

Transient transfections of 10 nM miR-450a mimics (Life technologies, AM17100) and 10 nM control scramble oligonucleotide (Life technologies) into DOK and SAS cells were performed using Lipofectamine RNAiMAX (Life technologies) according to the manufacturer’s instructions. For transfection of the other plasmids, cells were transiently transfected using Lipofectamine 2000 (Invitrogen, CA, USA) according to the manufacturer’s protocol.

### Adhesion and invasion assay

1x10^4^ of DOK cells or 2x10^4^ of SAS cells were seeded into the fibronectin (2 mg/ml; Corning, 356008) or matrigel^™^ (2 mg/ml; BD Biosciences) pre-coated 96-well plate, and incubated at 37°C for 1 h. After rinsed, attached cells were stained with 0.1% crystal violet and evaluated by measuring the absorbance at 595 nm in a Microplate reader (Molecular Devices, CA, USA). Invasion assays were performed as previously described [20]. Briefly, the invasion ability was determined using 24-well insert-based assays (BD Biosciences, Franklin Lakes, NJ). The upper insert, with 8 μm pore size, was coated with a density of 40 μg/well of Matrigel Basement Membrane Matrix (BD Biosciences). 2.5 x 104 cells were suspended in medium containing 10% NuSerum, and then added onto the upper insert. After incubating for 24 hours at 37°C, the cells that invaded through the Fluoro-Blok membrane were stained with propidium iodine, and fluorescence images were taken. The invasive cell numbers were then counted with Analytical Imaging Station software package (Imaging Research, Ontario, Canada).

### Luciferase reporter assay

DOK and SAS cells were transfected with100 ng of TMEM182 3′-UTR wild-type (WT) or truncated (DEL) pmirGLO reporter plasmid and transfected with 10 nM of miR-450a mimics or control oligonucleotide (scramble) with Lipofectamine 2000 according to the manufacturer’s instructions. The activity of luciferases was determined at 48 h post transfection with Dual Luciferase Reporter Assay System (Promega, USA) as described by the manufacturer’s protocol. Luminometry readings were obtained using an Orion L luminometer (Berthold).

### Statistical analysis

All quantitative results were from at least three independent experiments and reported as the mean±SEM. A linear correlation and Pearson correlation were used to investigate the association between 2 variables. Differences of various groups were assessed by one-way analysis of variance (ANOVA) and paired Student’s t-tests, unless otherwise stated. Recurrent analysis was calculated using Pearson Χ^2^ test. Kaplan-Meier method was using to calculate prognostic values with the log-rank test. All statistic values were carried out using GraphPad Prism V. 4.01 (San Diego, CA). *P*<0.05 were considered statistically significant and represented as *P<0.05; **P<0.01; ***P<0.001.

## Results

### miR-450a mediates cellular adhesion and invasion in OSCC

**Fig 1:**
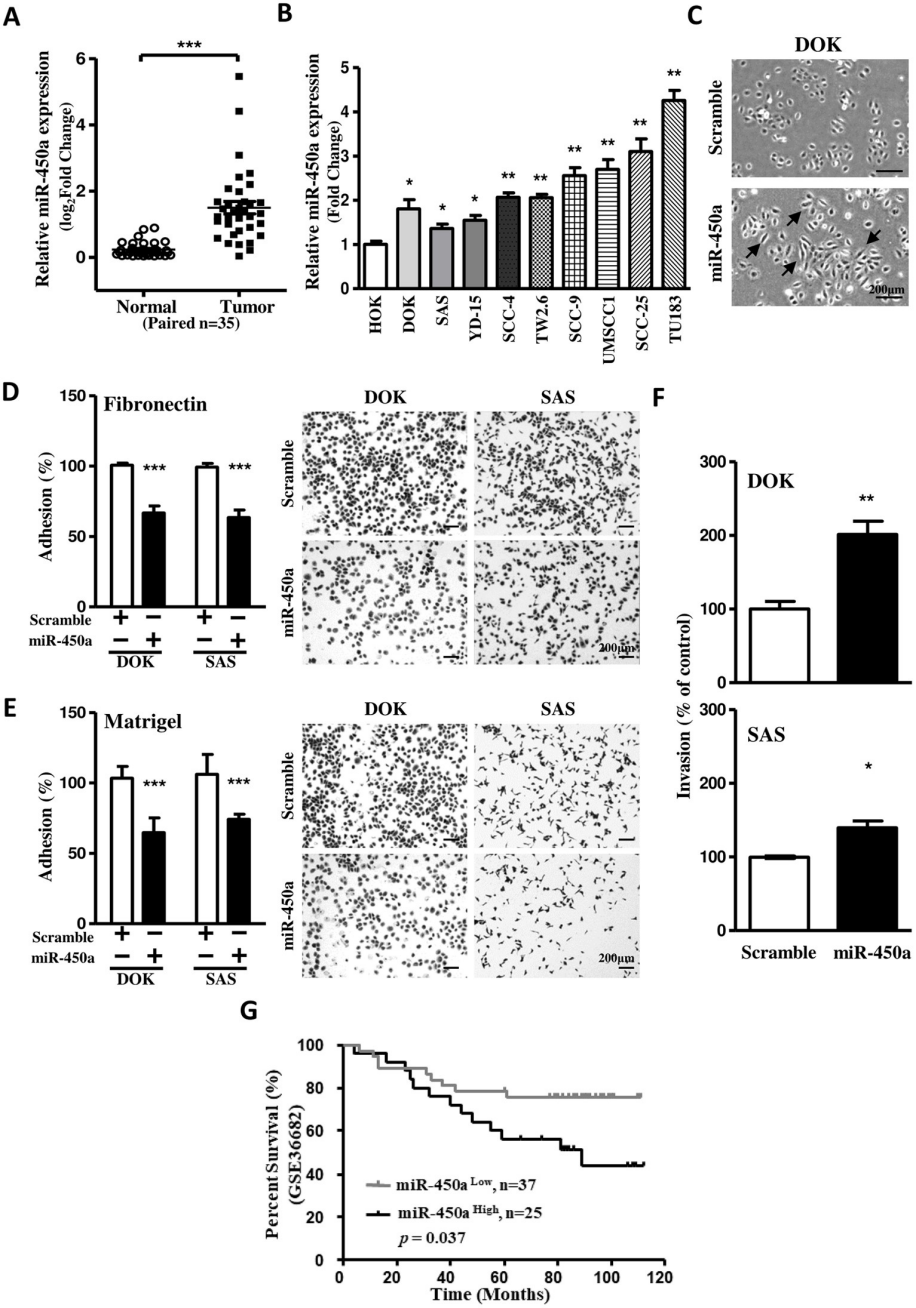
Up-regulated miR-450a impairs cell adhesion and enhances invasion of OSCC. **(A-B)** The expression levels of miR-450a in human OSCC clinical specimens (n = 35) and human OSCC cell lines (n = 9) were measured and normalized to RNU44. **(C)** Presentative DOK morphology changes under the transfection of miR-450a mimic and control (scramble). Arrowhead indicates that a morphological change from a rounded shape into a spindle-like shape. **(D-E)** Cell adhesion assay in DOK and SAS cells transfected with miR-450a mimic and scramble control on fibronectin and matrigel were stained and quantified as described in methods. Bars in the right lower corners of all photos are equivalent to 200 μm. **(F)** Transwell invasion assays were used to measure the effect of miR-450a in DOK and SAS cells after 48 hrs transfection. **(G)** Kaplan-Meier survival plot for miR-450a expression in HNSCC patients. The survival curves were analyzed in GSE36682 cohort (n = 62). Presentative images were performed from at least three independent experiments. Data was represented as mean±SEM; **P<0.01; ***P<0.001.

To examine the expression pattern of miR-450a which screened from OSCC microarray data [10], we used quantitative real-time PCR to measure the miR-450a expression in another thirty-five clinical OSCC specimens. We found that the expression levels of miR-450a were significantly higher in 35 OSCC tumors compared with their corresponding normal samples (p<0.0001, Fig 1A). Similar results were observed in OSCC cell lines. The expression levels of miR-450a in these OSCC cell lines were significantly higher than the normal human oral keratinocyte HOK (Fig 1B). To study the potential functions of miR-450a in OSCC, we introduced DOK cells with miR-450a mimics. Specifically, we observed a morphological change, from a rounded shape into a spindle-like shape, in comparison with the scramble control transfectants (Fig 1C). According to the alteration of cell morphology, we hypothesized that miR-450a might regulate cell adhesion. To test this hypothesis, DOK and SAS cells were subjected to adhesion assays on various components of the extracellular matrix (ECM). Overexpression of miR-450a in DOK and SAS cells showed significant decreases in adhesion ability on fibronectin and matrigel (Fig 1D and 1E). Furthermore, overexpressed miR-450a increased OSCC cells invasion capacity (Fig 1F) and had a poor prognosis in HNSCC patients (Fig 1G). These results indicate that miR-450a may have oncogenic effects in OSCC cells. Augmented miR-450a reduced the cellular adhesion and consequently induced invasion in oral carcinogenesis.

### TMEM182 is directly targeted by miR-450a

**Fig 2:**
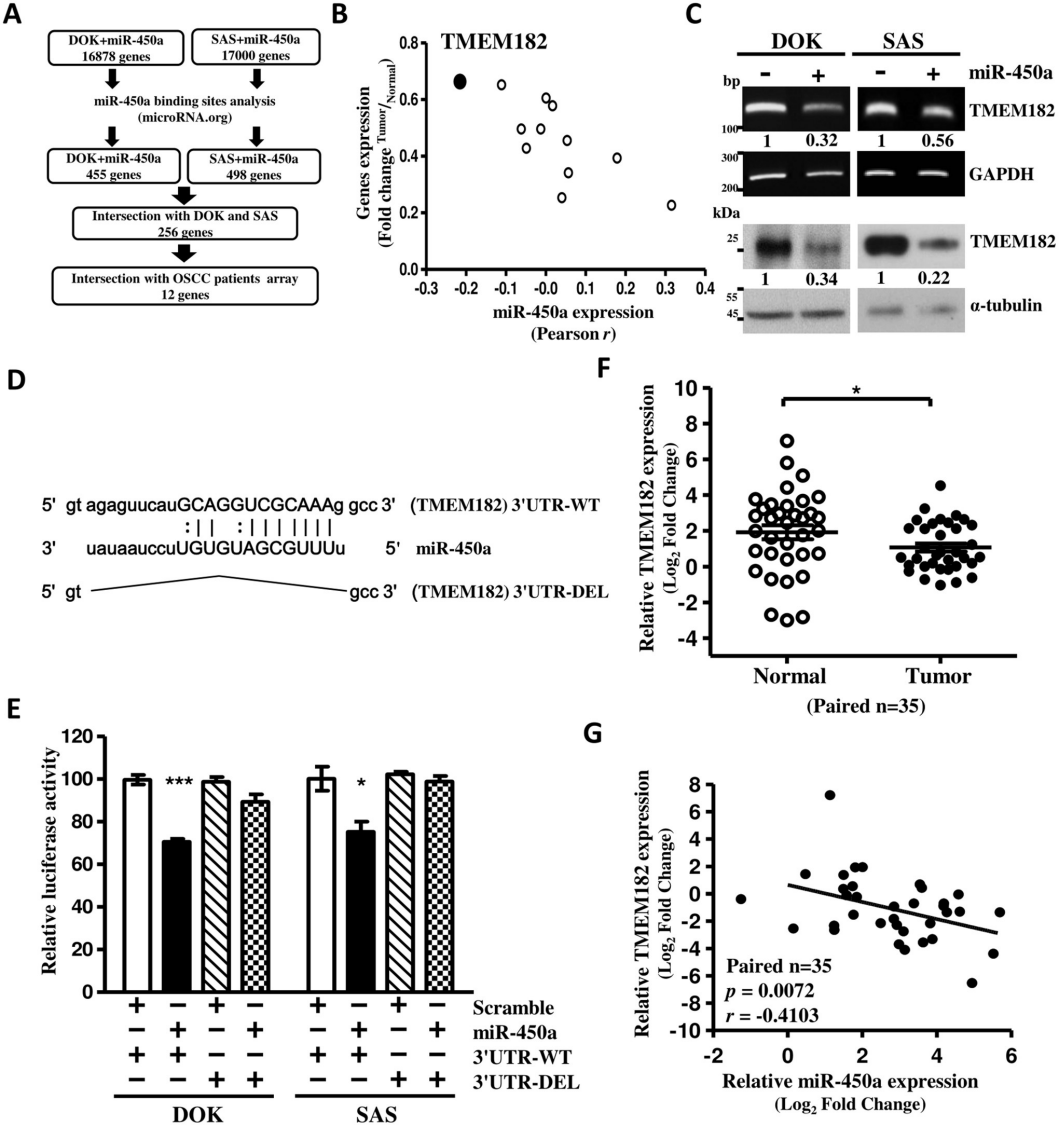
miR-450a decreases TMEM182 by directly targeting 3'-UTR. (A) Flowchart for *in silico* analysis of miR-450a-regulated genes from OSCC cell lines (DOK and SAS cells) and our previous OSCC clinical samples data (n = 40)(accession number GSE37991). **(B)** Twelve of miR-450a-targeted candidates were evaluated on the basis of down-regulated rates (fold change) and Pearson *r* correlation against miR-450a expression in previous OSCC clinical samples (n = 40) data. TMEM182 (black circle) presented the best negative correlation with miR-450a. **(C)** Levels of TMEM182 changes in DOK and SAS cells were assessed with RT-PCR and western blot after miR-450a mimics/scramble transfection for 48 hrs. Numerical values for band intensities are shown below the gels. The values were quantitated by densitometry and normalized to GAPDH or α-tubulin. **(D)** Schematic representation of predicted miR-450a binding sequence in the 3'-UTR of TMEM182 with wild-type form (3'UTR-WT), and with miR-450a binding site deleted form (3'UTR-DEL). **(E)** miR-450a regulated TMEM182 3'-UTRluciferase activities of 3'-UTR-WTor 3'-UTR-DEL in DOK and SAS cells after 48 hrs transfection as described in panel. The relative luciferase activities are the ratios of Renilla luciferase normalized to scramble. **(F)** Levels of TMEM182 in OSCC human samples (n = 35) was assessed with qPCR. (Student’s t test, *p*<0.05). **G**, The Pearson *r* correlation between miR-450a and TMEM182 levels in OSCC patients (n = 35) by qPCR analysis. MiR-450a expression was normalized to RNU44 and TMEM182 expression was normalized to GAPDH. Data was represented as mean±SEM; *P<0.05; **P<0.01; ***P<0.001.

In order to determine the downstream target genes of oncogenic miR-450a in OSCC, we performed genome-wide gene expression analysis using miR-450a transfected DOK and SAS cells. Our strategy for collection of miR-450a downstream target genes is presented in Fig 2A. Compared with control cells, a total of 16878 and 17000 genes were downregulated in miR-450a transfected DOK and SAS cells, respectively. These genes were then analyzed the putative binding sites of miR-450a in their 3’-UTR by microRNA.org database. Through this step, we found 455 genes in DOK group and 498 genes in SAS group with miR-450a binding sites in their 3’-UTR, respectively. Combining the results of these two sets, we identified 256 of common genes which were not only downregulated in miR-450a transfected DOK and SAS cells, but also with miR-450a binding sites. Next, to verify the clinical significance of these genes, we intersected the 256 genes with 40 pairs of OSCC patients which expression data deposited in GEO (accession number GSE37991) by our previously study [10]. Finally, we got a set of 12 genes which were downregulated in OSCC tumors compared with their corresponding normal samples (S3 Table). However, when we correlated these 12 genes with miR-450a expression, we found that 7 genes were positively correlated with miR450a expression in OSCC tumors. This result doesn’t meet our expectation. Only 5 genes showed a negative correlation with miR-450a expression in OSCC patients. Among these 5 genes, TMEM182 is the best negatively correlation with miR-450a (S3 Table and Fig 2B). Therefore, we focused on the TMEM182 as a possible target gene regulated by miR-450a in OSCC and for further study.

Overexpression of miR-450a mimics in DOK and SAS cells decreased TMEM182 expression at mRNA and protein levels compared with miR-scramble control in OSCC cells (Fig 2C). To confirm whether TMEM182 is a direct targeted by miR-450a, we performed a luciferase reporter assays using a vector encoding the partial sequence of the 3’-UTR of TMEM182 either including (WT) or excluding (DEL) the miR-450a binding sites (Fig 2D). We observed that luciferase intensity was significantly reduced by transfection of wild-type 3’-UTR of TMEM182, but not in cells transfected with the TMEM182 3’-UTR containing the deleted miR-450a binding sites (Fig 2E). We also analyzed the TMEM182 mRNA levels using the same clinical OSCC specimens in Fig 1a and found that the TMEM182 expression was lower in OSCC tissues than in their corresponding normal samples (Fig 2F). To consolidate our findings, we correlated the expression levels of miR-450a and TMEM182 in clinical OSCC specimens and found a strong inverse correlation between the miR-450a and TMEM182 (Fig 2G). Taken together, our results demonstrated that TMEM182 is a direct target of miR-450a.

### TMEM182 regulates cell adhesion and invasion in OSCC cells

**Fig 3:**
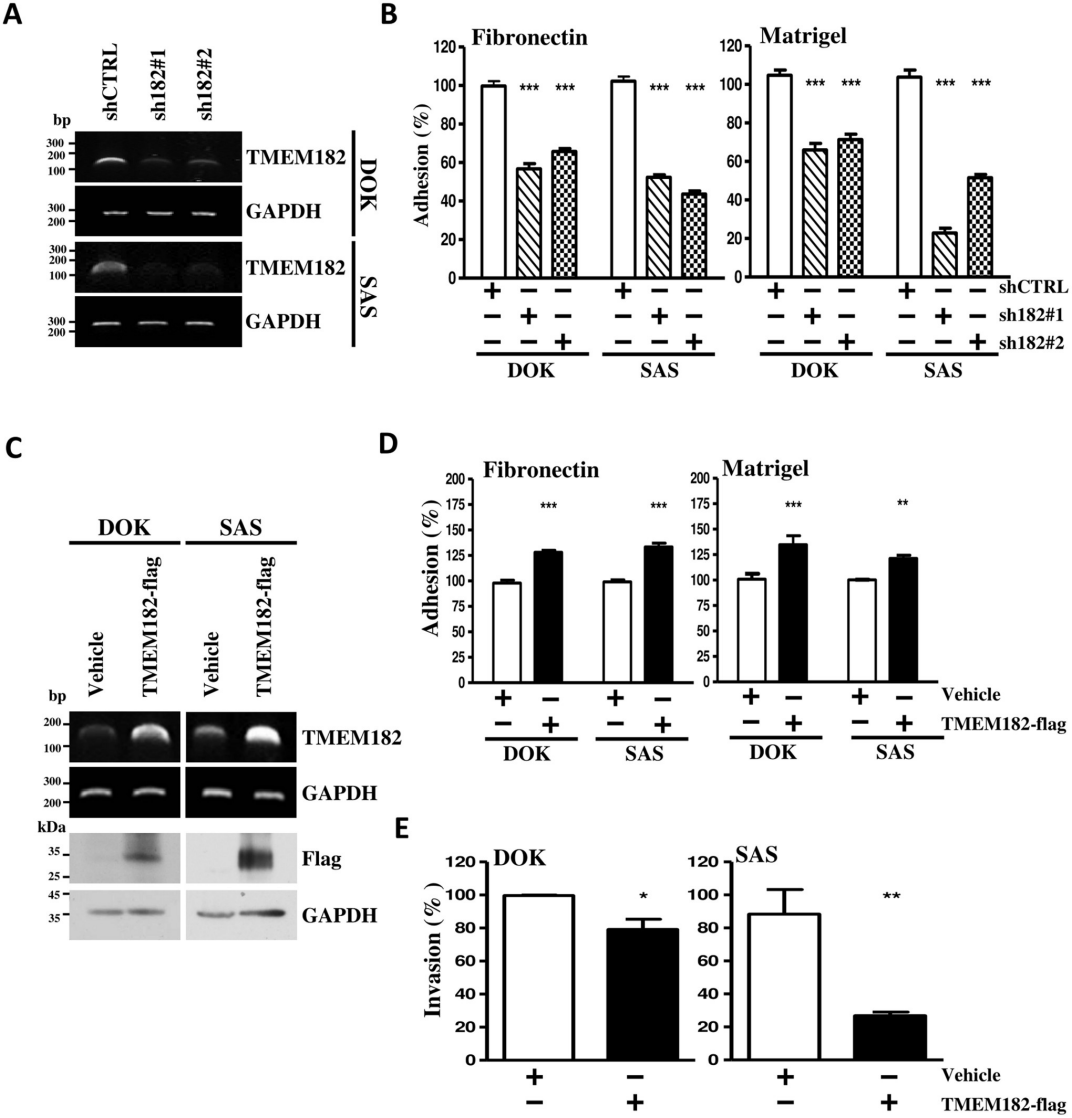
TMEM182 decreases OSCC cell motility. **(A)** RT-PCR analyses were performed to detect mRNA expression level of TMEM182 in DOK and SAS cells transfected withTMEM182 knockdown clones-shRNA clone 1 (sh182#1), clone 2 (sh182#2), or empty vector (shCTRL). GAPDH was used as a loading control. **(B)** Suppression of cell adhesive ability was found in TMEM182-knockdown cells towards to fibronectin and matrigel. **(C)** RT-PCR and Western blot analyses measured the levels of TMEM182 in vehicle or TMEM182-flag transfected cells. GAPDH and α-tubulin were used as loading controls. **(D-E)** Cell adhesion and invasion analyses of TMEM182 overexpression in DOK and SAS were measured. Data was represented as mean±SEM; *P<0.05; **P<0.01; ***P<0.001.

To determine the effect of TMEM182 on cell adhesion, we performed loss-of-function studies by specific shRNAs in DOK and SAS cells. Levels of TMEM182 mRNA were repressed by shTMEM182 transfectants (Fig 3A). TMEM182 knockdown in DOK and SAS significantly reduced their cell adhesion ability towards to fibronectin and matrigel (Fig 3B). Whereas, overexpression of TMEM182 in DOK and SAS cells (Fig 3C) not only promoted the cell adhesion ability (Fig 3D), but also suppressed the invasion of DOK and SAS cells (Fig 3E). Our data suggested that TMEM182 may play a role in regulating OSCC invasion and adhesion abilities.

### MiR-450a enhances cell adhesion throughTMEM182 downregulation

**Fig 4:**
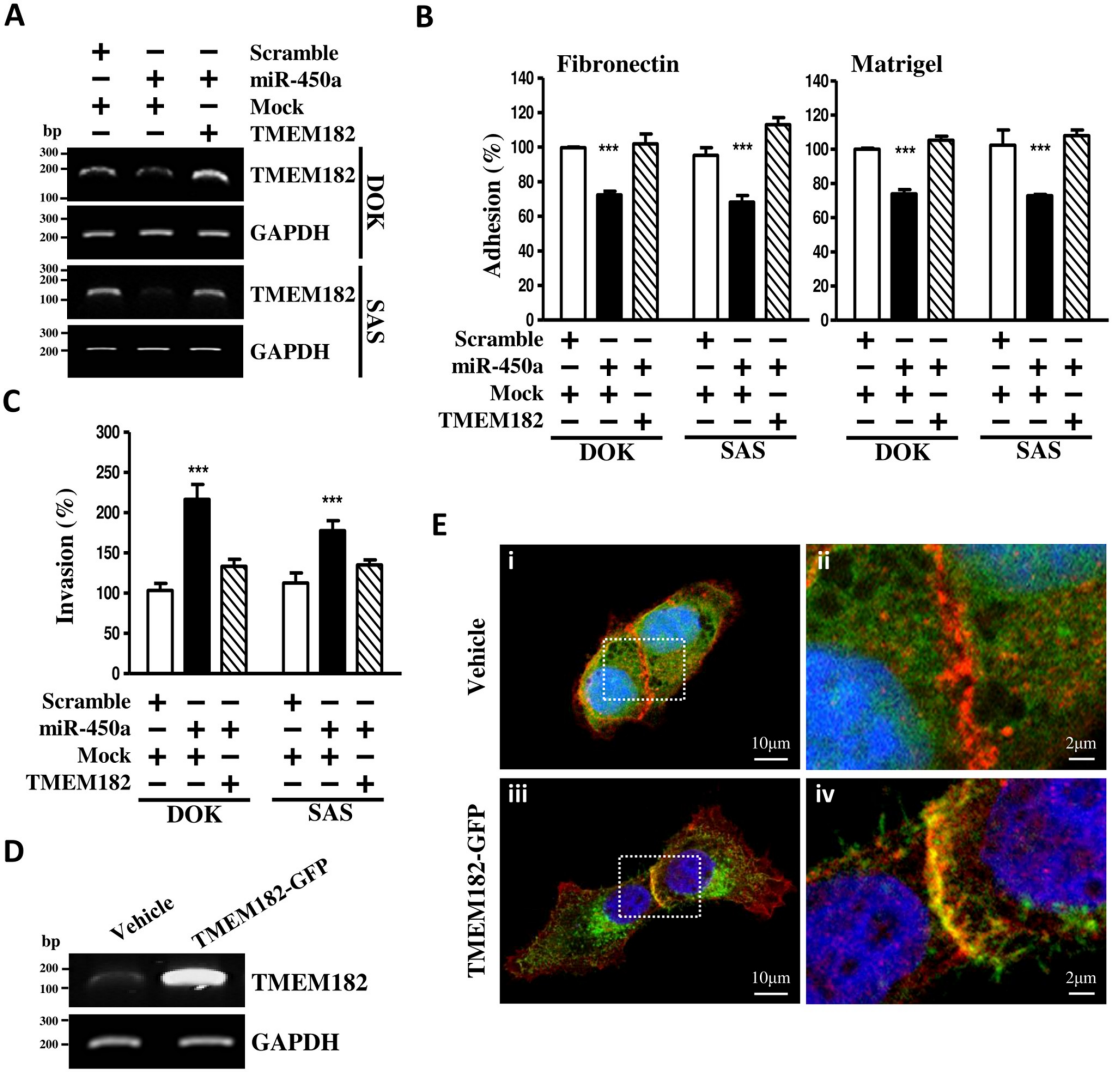
Overexpression of TMEM182 renders human OSCC cells resistance to miR-450a-decreased cell adhesion. **(A)** Changes of TMEM182 levels in OSCC cells transfected with control miRNA (scramble)/miR-450a and pCDH-CMV-GFP puro+ (vehicle)/TMEM182 (TMEM182-flag) were assessed by RT-PCR as described in panel. GAPDH was used as a loading control. **(B)** Cell adhesion assays of OSCC cells transfected with scramble/miR-450a and vehicle/TMEM182-flag were as described above. **(C)** Transwell invasion assays were used to measure the effect of scramble/miR-450a and vehicle/TMEM182-flag in DOK and SAS cells after 48 hrs transfection. (D) TMEM182 overexpression in SAS cells was confirmed with RT-PCR after transfection of GFP-linked TMEM182 (TMEM182-GFP) and empty vector pEGFPN1 (vehicle). **(E)** Representative epifluorescence images of SAS cells transiently transfected with TMEM182-GFP/ Vehicle and co-immunolabeled endogenous E-cadherin. Vehicle (Green) expression was scattered inside of cells **(D-i, D-ii)**. Co-stained E-cadherin (Red), as a plasma membrane marker, was localized at the lateral membrane and intracellular junctional area. Nuclei were labeled with DAPI (Blue).TMEM182-driven GFP(green) was co-localized with E-cadherin (red) at the sites of cell-cell contact on the plasma membrane **(D-iii, D-iv)**. Original magnification, x400. Data was represented as mean±SEM; *P<0.05, **P<0.01, ***P<0.001.

Next, we tried to investigate whether miR-450a induced TMEM182 downregulation plays a major role on cell adhesion. For this purpose, we generated a 3’-UTR-lacking TMEM182 vector and transfected it alone or in combination with miR-450a in DOK and SAS cells. As expected, miR-450a decreased endogenous TMEM182 expression and restoration of 3’-UTR-lacking TMEM182 successfully rescued miR-450a-decreased endogenous TMEM182 expression (Fig 4A). Moreover, the restoration of 3’-UTR-lacking TMEM182 rescued the adhesion ability suppressed by miR-450a but suppressed the invasion ability induced by miR-450a (Fig 4B and 4C). Our results indicated that miR-450a-mediated TMEM182 function was crucial for regulating attachment ability of OSCC cells. On the basis of functional sequences annotations from NCBI database and Kyte-Doolittle hydrophobicity analysis (S1 Fig), there are four predictable hydrophobic regions which aligned in the protein sequences of TMEM182. This result implies that tmem182 may be a membrane protein. To prove that, GFP linked TMEM182 was generated to observe TMEM182 expression (Fig 4D) and location in cells (Fig 4E and S2 Fig). TMEM182 appeared at lateral membrane zones; particularly at cell-cell contact sites on the plasma membrane (Fig 4E). Taken together, these data demonstrate that miR-450a-mediated TMEM182 functions are crucial in regulating cell adhesion and invasion in OSCC cells.

### TNFα triggered-miR-450a attenuates TMEM182 expression in OSCC

**Fig 5:**
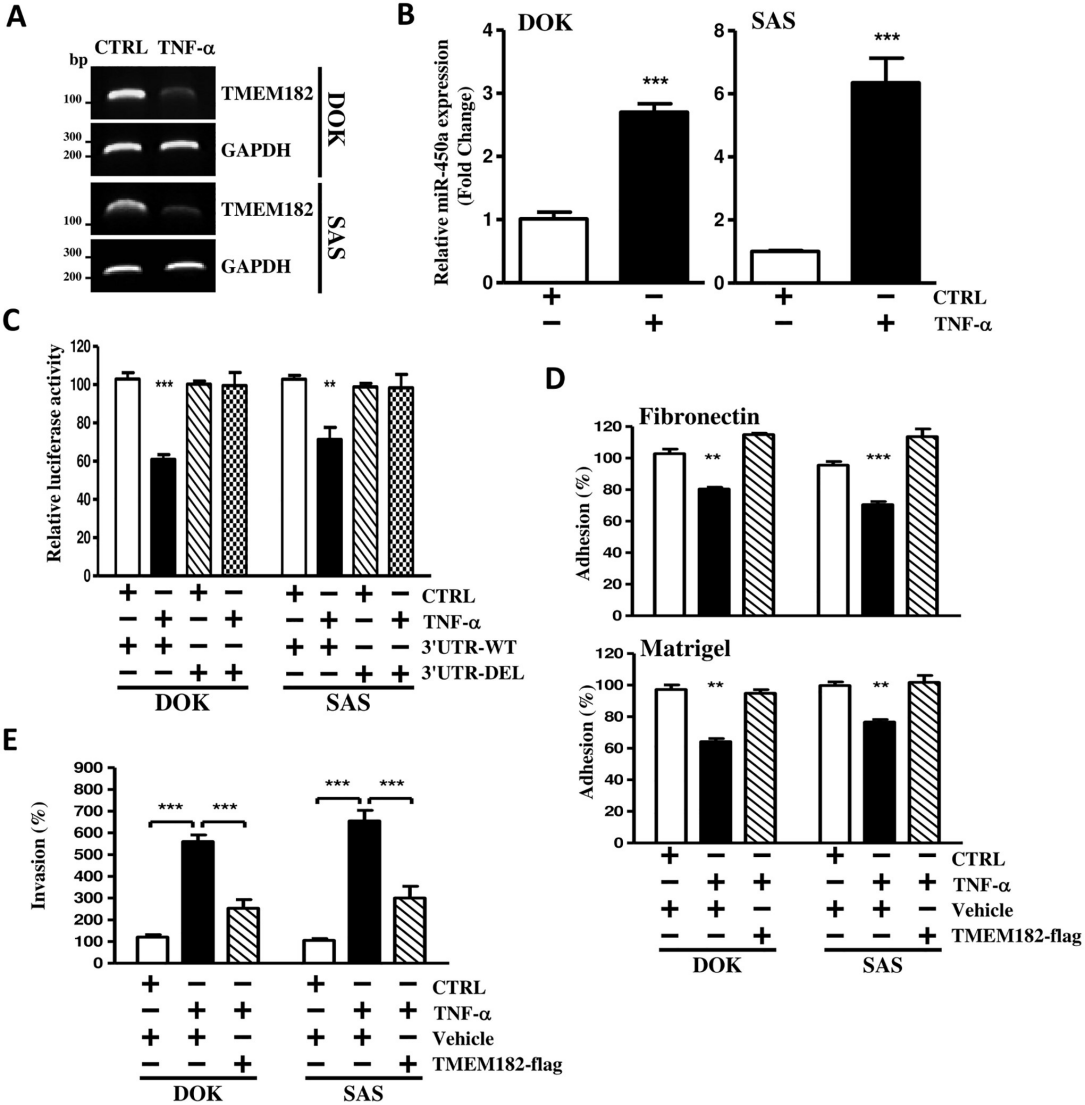
TMEM182 is down-regulated by miR-450a in response to TNF-α in OSCC cells. **(A)** Levels of TMEM182 in DOK and SAS cells treated with either ddH_2_O (CTRL) or TNF-αwere assessed with RT-PCR. GAPDH was used as a control. **(B)** miR-450a changes in DOK and SAS cells treated with or without TNF-α were measured with qPCR and normalized to RNU44. **(C)** Luciferase activity measured that TNF-α regulated TMEM182 through miR-450a binding site at 3'-UTR. Cells were transfected with TMEM182 3'-UTRs constructed either with wild-type (3'-UTR-WT) or miR-450a binding site truncated (3'-UTR-DEL), before TNF-α was added for 24 hrs as described in panel. Relative luciferase activities were the ratios of Renilla luciferase normalized to the wild-type control. **(D-E)** Cells were transfected with either empty vector pCDH-CMV-GFP puro+ (Vehicle) or TMEM182 (TMEM182-flag) followed by the stimulation with TNF-α for 24 hrs, and then, corresponding adhesion ability and invasion ability were measured. Data are represented as mean±SEM; **P<0.01; ***P<0.001.

Previous study has shown that TNF-α could downregulate TMEM182 transcript in 3T3-L1 adipocytes [21]. However, little is known about the underlying mechanism between TNF-α and TMEN182 in oral cancer. Thus, we hypothesized that miR-450a may involve in the TNF-α-induced TMEM182 downregulation. To address this, we first tested the ability of TNF-α on TMEM182 expression in OSCC cells. We found that TNF-α treatment indeed significantly reduced the mRNA level of TMEM182 in DOK and SAS cells (Fig 5A). Simultaneously, we also observed that TNF-α treatment not only induced miR-450a expression (Fig 5B) but also decreased TMEM182 expression by targeting miR-450a binding site at TMEM182 3'-UTR (Fig 5C). However, transfection of TMEM182 3’-UTR containing the deleted miR-450a binding sites (3’UTR-DEL) abolished the TNF-α-induced miR-450a binding to TMEM182 3’-UTR (Fig 5C), indicated that TMEM182 is directly targeted by miR-450a upon TNF-α treatment. Furthermore, overexpression of TMEM182 reversed the cell adhesion reduced by TNF-α treatment and suppressed the invasion ability induced by TNF-α (Fig 5D and 5E). These evidences showed that TMEM182 was mediated by miR-450a in response to TNF-α.

### TNFα induces miR-450a expression via endogenous ERK and NFκB pathways

**Fig 6:**
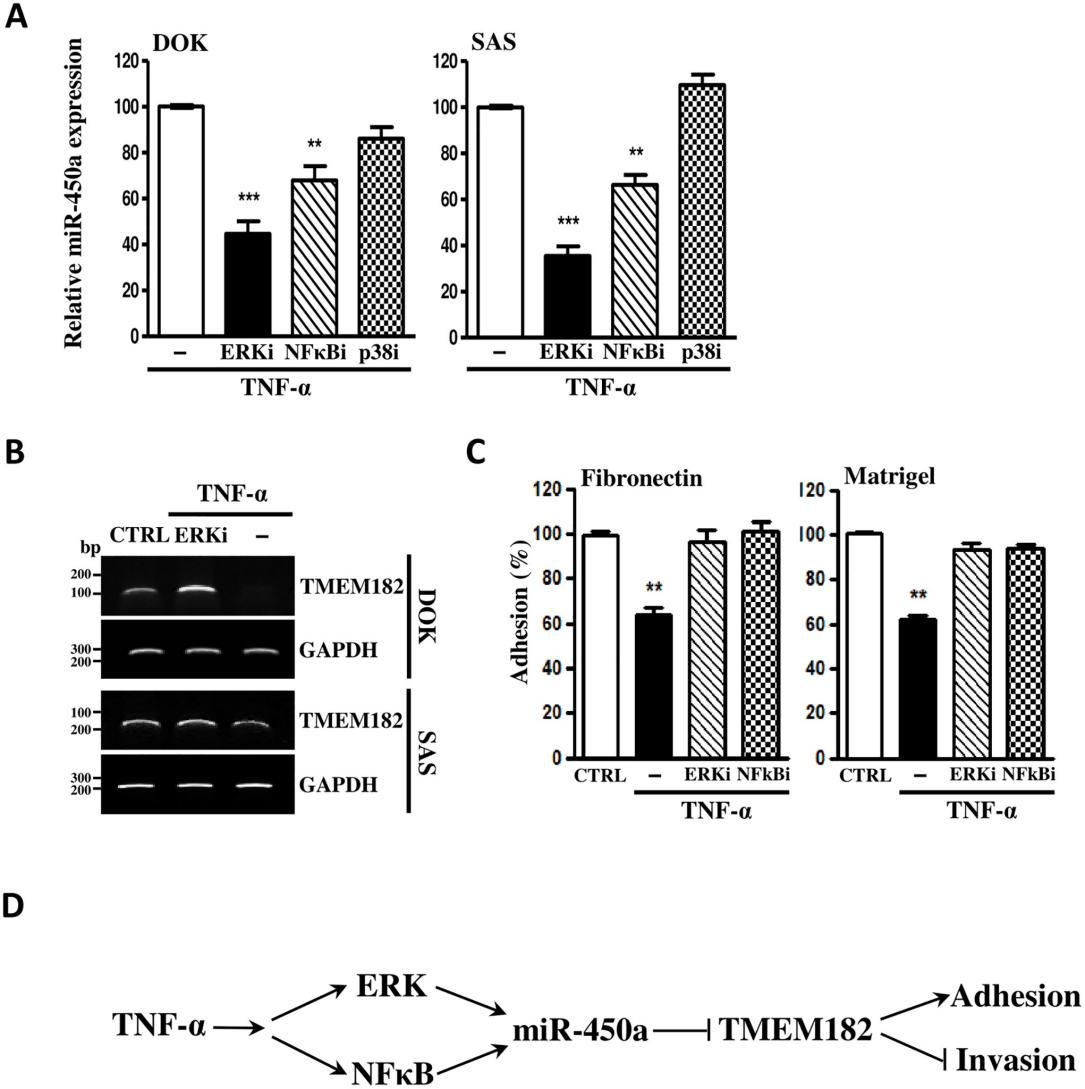
TNFα upregulates miR-450a versus ERK and NFκB pathways to inhibit TMEM182 stabilized OSCC cells mobility. **(A)** OSCC cells were subjected into negative control DMSO (-), ERK inhibitor (ERKi), NF-κB inhibitor (NF-κBi), or p38 inhibitor (p38i) as described above. After 6 hrs, TNF-α and ddH_2_O (negative control) were added as indicated treatments. Twenty-four hrs later, miR-450a levels were evaluated with qPCR. Levels of miR-450a in described conditions were standardized to negative controls (DMSO + ddH_2_O one). TNF-α only was used as reference. **(B)** RT-PCR analyses revealed that TNF-α suppressed TMEM182 expression in human OSCC cells was restored by ERKi. ddH_2_O and DMSO (-) were used as negative controls. GAPDH was used as the loading control. **(C)** Cell adhesion analyses revealed that ERKi or NF-κBi pretreated SAS cells successfully abolished TNF-α reduced cell adhesion ability. **(D)** TNF-α-induced miR-450a repressed TMEM182 expression to promote tumor malignancy through both intrinsic ERK and NF-κB pathways. Results were represented as mean±SEM;**P<0.01, ***P<0.001.

Some pathways, such as ERK1/2, NF-κB, or p38, are known to be the principle downstream signaling of TNF-α [22]. To further delineate the mechanisms of TNF-α-signaling to miR-450a expression, we examined the relevance of ERK1/2, NF-κB, or p38 signaling activated by TNF-α. As shown in Fig 6A, ERK inhibitor (PD98059) and NF-κB inhibitor (NF-κBi), but not p38 inhibitor (SB20219), significantly blocked the TNF-α-induced miR-450a expression in both DOK and SAS cell lines. Under this condition, ERK inhibitor (PD98059) and NF-κB inhibitor (NF-κBi) can effectively inhibit the ERK1/2 and NFkB activation (S3A Fig). Furthermore, ERK inhibitor (PD98059) and NF-κB inhibitor (NF-κBi) not only rescued the TNF-α-induced TMEM182 downexpression (Fig 6B) but also rescued the TNF-α-induced adhesion reduction (Fig 6C). Otherwise, we found that the activities of ERK and NFkB were initiated at 10 mins after TNF-α treatment and the activity could sustain to 60 mins. However, the level of miR-450a does not rise significantly until 30 mins (S3B and S3C Fig). Therefore, we think that miR-450a expression is an immediate downstream target of ERK/NFkB signaling pathway. Our data suggested that TNF-α-induced miR-450a and reduced cell adhesion by decreasing TMEM182 via intrinsic ERK1/2 and NF-κB pathways (Fig 6D).

## Discussion

MiR-450a is identified as one of prognostic markers for adrenocortical tumor, aristolochic acid nephropathy and type 2 diabetes [23–25]. Emerging data indicated that miR-450a controls a divergent function on the osteoblastic differentiation of human mesenchymal stem cells by targeting STAT1 and rats’ adipogenesis by targeting WISP2 [26, 27]. However, there are no data to suggest a connection between miR-450a and oral cancer in recent investigations. In this study, gain-of function approach indicated that miR-450a restoration significantly inhibited cell adhesion and enhanced invasion ability in DOK and SAS cells, suggesting miR-450a is an onco-miR and play an important role in OSCC cellular adhesion on matrix. For elucidation the molecular mechanisms and identification the putative targets of miR-450a in OSCC, we performed genome-wide gene expression analysis using miR-450a transfected DOK and SAS cells. In silico analysis the expression signatures of miR-450a transfectants in OSCC cells and OSCC patients, we found that twelve genes were downregulation in clinical specimens and that they were candidate targets of miR-450a. Among these genes, transmembrane protein TMEM182 showed a best inverse correlation with miR-450a. Downregulating TMEM182 using shRNA suppressed cell adhesion in a manner comparable to miR-450a overexpression. Moreover, the restoration of TMEM182 potently rescued the adhesion ability suppressed by miR-450a, suggesting that miR-450a mainly acts as a novel regulator in mediating OSCC adhesion by targeting membrane protein TMEM182.

*TMEM182* gene encodes an entirely 229-amino-acid protein, which is predicted to consist of four putative membrane-spanning regions (S1 Fig). It is highly evolutionary conserved among different species [21]. Even TMEM182 plays important roles in adipogenesis, myogenesis, and glaucoma [18, 21, 28], however, its working mechanisms were still unknown. Dissolution of junctional connection, detachment to ECM, and migration are key steps of OSCC loco-regional invasion [29–31]. Our findings demonstrated that overexpression of TMEM182 increased OSCC adhesive ability and restrained its invasiveness. Moreover, restoration of TMEM182 completely rescued the cellular attachments suppressed by miR-450a in vitro. Thus, decreased cell-matrix adhesion might enhanced the cellular contraction and thereby facilitate tumor migration and invasion. On the other hand, disassembly of cell-cell interaction is occurrence at the early stage of OSCC invasion [30]. Cell adhesion molecules, such as integrin, cadherin family, and immunoglobulin superfamily, play a role in cell-cell interactions and involved in the process of tumor invasion and metastases [30, 32–34]. Loss of these cell adhesion molecules is associated closely with invasion and could be used for the prognostic purposes in oral cancer [35–38]. In this study, immunofluorescence data demonstrates that TMEM182 appeared at lateral membrane zones; particularly at cell-cell contact sites on the cell membrane. These results suggest that TMEM182 may play a role in cell-cell interaction and cell-extracellular matrix adhesion as a result of involving in the process of tumor invasion. However, little studies have described the functions of TMEM182 or their relationship between cell-cell interaction and cell-extracellular matrix adhesion. The detail mechanisms remain to be elucidated.

Recent studies present that inflammatory factors, including TNF-α, are potential prognostic biomarkers for OSCC [39, 40]. Our findings supported that TNF-α activated endogenous ERK1/2 and NF-κB pathways to induce miR-450a expression. Current studies reveal that TNF-α induced EMT to promote OSCC invasion through NF-κB pathway by targeting at well-known Snail and Id2 [41–43]. It is worth noting that the miR-450a expression induced by TNF-α primarily through ERK1/2 activation rather than through NF-kB pathway. As a consequence, TMEM182 was downregulated by miR-450a to increase OSCC cells invasion.

To our knowledge, this study is the first to describe the roles of ERK1/2 and NF-κB in TNF-α-induced miR-450a expression in human OSCC. Upregulation of miR-450a could reduce cellular adhesion to matrix by targeting TMEM182 and enhance tumor invasion. Therefore, TNF-α/miR-450a/TMEM182 signaling axis may be a novel potential target for clinical intervention in oral cancer.

## Supporting information

S1 Fig Hydrophobic region prediction of TMEM182. Protein length of human TMEM182 precursor (229a.a.) was analyzed by ExPasy database. Total four transmembrane regions were indicated by Arabic numbers in order and Alphabet letter S. S indicated as a potential signal sequence.
(PDF)

S2 Fig Fluorescence of TMEM182 and E-cadherin in OSCC cells. Epifluorescence images of SAS cells transiently transfected with TMEM182-GFP/ Vehicle and co-immunolabeled endogenous E-cadherin with divided channels. **(A)** Vehicle (Green) expression was spread all over the cells. **(B, E)** Membrane marker Ecadherin (Red) was localized at intracellular junctional areas. **(C, F)** Nuclei were labeled with DAPI (Blue). **(D)** TMEM182-driven GFP (green) located at cell-cell contact sites on the lateral membrane and endoplasmic reticulum. Scale bars were indicated in panel.
(PDF)

S3 Fig Effects of ERK and NFκB pathways on TNF-α. **(A)** OSCC cells were pre-incubated with either DMSO vehicle (-), ERK inhibitor (ERKi, 30 μM), NF-κB inhibitor (NF-κBi, 10 μM) for 6 h and then treated with 10 ng/ml of TNF-α for another 24 hrs, followed by measurements of TMEM182 expression by western blotting. GAPDH was used as an internal control. **(B)** Western blotting analysis of ERK and NFκB activity after TNF-α treatment in SAS cells at indicated time. GAPDH was used as an internal control. (C) miR-450a expression level in SAS cells treated with TNF-α using qRT-PCR and normalized to RNU44. Results were represented as mean±SEM;**P<0.01, ***P<0.001.
(PDF)

S1 Table Patients’ cinicalpathological analysis. (DOC)

S2 Table Sequences of primers. (DOCX)

S3 Table Twelve of candidate genes downregulated by miR-450a. (DOC)
