# Supplementary figures and images for: TNF-α-induced miR-450a mediates TMEM182 expression to promote oral squamous cell carcinoma motility
Source: PLoS One. 2019 Mar 20;14(3):e0213463. doi: 10.1371/journal.pone.0213463 (PMC6426234; doi:10.1371/journal.pone.0213463)

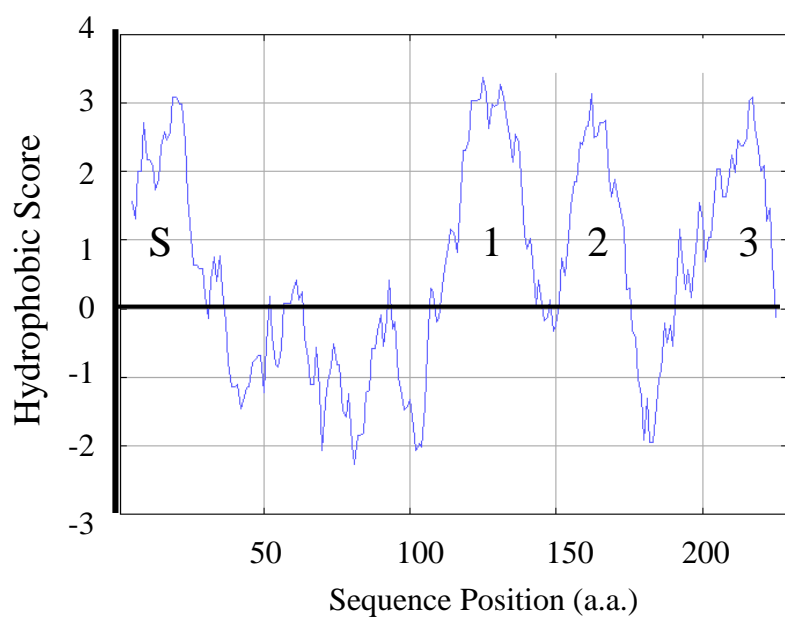

Supplement: S1 Fig — Protein length of human TMEM182 precursor (229a.a.) was analyzed by ExPasy database. Total four transmembrane regions were indicated by Arabic numbers in order and Alphabet letter S. S indicated as a potential signal sequence. (PDF) [file pone.0213463.s001.pdf]

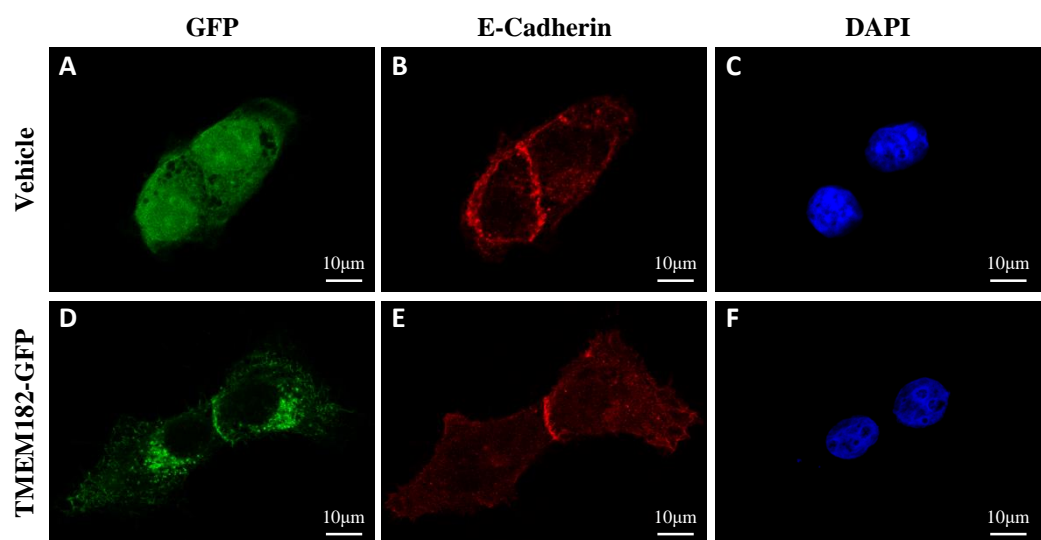

Supplement: S2 Fig — Epifluorescence images of SAS cells transiently transfected with TMEM182-GFP/ Vehicle and co-immunolabeled endogenous E-cadherin with divided channels. (A) Vehicle (Green) expression was spread all over the cells. (B, E) Membrane marker Ecadherin (Red) was localized at intracellular junctional areas. (C, F) Nuclei were labeled with DAPI (Blue). (D) TMEM182-driven GFP (green) located at cell-cell contact sites on the lateral membrane and endoplasmic reticulum. Scale bars were indicated in panel. (PDF) [file pone.0213463.s002.pdf]

**A**

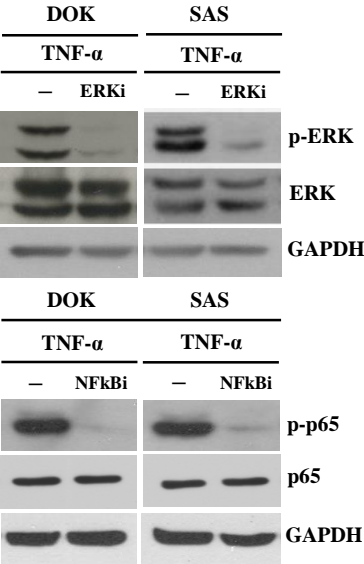

**B**

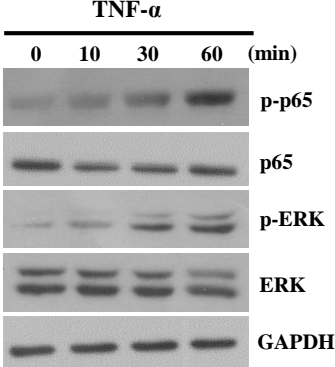

**C**

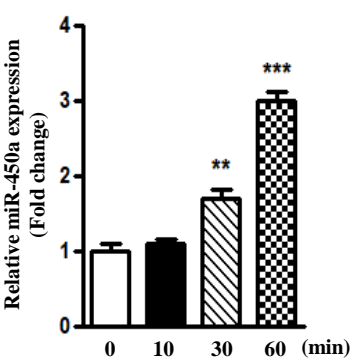

Supplement: S3 Fig — (A) OSCC cells were pre-incubated with either DMSO vehicle (-), ERK inhibitor (ERKi, 30 μM), NF-κB inhibitor (NF-κBi, 10 μM) for 6 h and then treated with 10 ng/ml of TNF-α for another 24 hrs, followed by measurements of TMEM182 expression by western blotting. GAPDH was used as an internal control. (B) Western blotting analysis of ERK and NFκB activity after TNF-α treatment in SAS cells at indicated time. GAPDH was used as an internal control. (C) miR-450a expression level in SAS cells treated with TNF-α using qRT-PCR and normalized to RNU44. Results were represented as mean±SEM;**P<0.01, ***P<0.001. (PDF) [file pone.0213463.s003.pdf]
