# Supplementary material for: TNF-α-induced miR-450a mediates TMEM182 expression to promote oral squamous cell carcinoma motility
Source: PLoS One. 2019 Mar 20;14(3):e0213463. doi: 10.1371/journal.pone.0213463 (PMC6426234; doi:10.1371/journal.pone.0213463)
Supplement: S1 Table — (DOC) [file pone.0213463.s004.doc]

| **Table S1. Patients' cinicalpathological analysis.** | | | | | |  |  |
| --- | --- | --- | --- | --- | --- | --- | --- |
| **Characteristics** | **Numbers (%)** | **Fold change of miR-450a^a^**  **log_2_ ratio** | | | | ***P*-value^b^** |  |
| ***Gender*** | | | | | | |  |
| Male | 32 (91.43) | 2.89 | | | | 0.35 |  |
| Female | 3 (8.57) | 2.99 | | | |  |
| ***Age (years)*** | | | | | | |  |
| Median (s.d.)**^c^** | 53 yrs (8.74) | - | | | |  |  |
| Range^##^ | 36-71yrs | - | | | |  |  |
| < Median (53yrs) | 17 (48.57) | 3.12 | | | | **0.03 ^*1^** |  |
| ≥Median (53yrs) | 18 (51.43) | 2 | | | |  |
| ***Localization*** | | | | | | |  |
| Buccal | 12 (34.29) | 1.85 | | | |  |  |
| Gingiva | 6 (17.14) | 2.56 | | | |  |  |
| Lip | 1 (2.86) | 1.25 | | | |  |  |
| Oral floor | 3 (8.57) | 3.63 | | | |  |  |
| Tongue | 13 (37.14) | 2.99 | | | |  |  |
| ***TNM stage*^d^** | | | | | | |  |
| T (Primary tumor) |  |  | | | |  |  |
| 1 | 3 (8.57) | 4.18 | | | | **0.026^*1^** |  |
| 2, 3, 4A | 32 (91.43) | 2.85 | | | |  |
| N (Regional lymph nodes) |  |  | | | |  |  |
| non | 22 (62.86) | 3.12 | | | | 0.38 |  |
| ≥1 | 13 (37.14) | 2.85 | | | |  |
| M (Distant metastasis) |  |  | | | |  |  |
| non | 22 (62.86) | 3.12 | | | | 0.37 |  |
| ≥1 | 13 (37.14) | 2.85 | | | |  |
| ^a^ Fold change was 2^(-ΔΔCt)^, ΔΔCt was the difference between Tumor and corresponding adjacent normal regions. | | | | | | | |
| ^b^ Students' t test analyses, P-value (one-tail, 2 types), significant *p* value was identified as *p*<0.05, labeled as *1. | | | | | | | |
| ^c^ Arabic numbers indicated patient's age in years. | | | | |  | | |
| ^d^ Tumor size, lymph node involvement, and distal metastasis were determinded according to months. the definition of the AJCC TNM staging system of HNSCC (12). Follo-up occurred for upto100. | | | | | | | |
|  | | | | | | | |
|  | | |  |  |  | | |
