# Supplementary material for: TNF-α-induced miR-450a mediates TMEM182 expression to promote oral squamous cell carcinoma motility
Source: PLoS One. 2019 Mar 20;14(3):e0213463. doi: 10.1371/journal.pone.0213463 (PMC6426234; doi:10.1371/journal.pone.0213463)
Supplement: S2 Table — (DOCX) [file pone.0213463.s005.docx]

| **Table S2**. **Sequences of primers** |
| --- |
| \| ***for gene expression*** \| \| \| --- \| --- \| \| TMEM182-F \| 5'-TGGTACACCAATCAGCCACC-3' \| \| TMEM182-R \| 5'-GCAGAGTCATAGGAGGTCGAG T-3' \| \| GAPDH-F \| 5'-GAAGGTGAAGGTCGGAGT-3' \| \| GAPDH-R \| 5'-GAAGATGGTGATGGGATTTC-3' \| \|  \|  \| \| ***for miRNA expression*** \| \| \| Universal Reverse \| 5'-GTGGAGGGTCCGAGGT-3' \| \| miR-450a-RT \| 5'-GTTGGCTCTGGTGCAGGGTCCGAGGTATTCGCACCA  GAGCCAACATATTA-3' \| \| miR-450a-F \| 5'-CGGTTTTGCGATGTGTTCC-3' \| \| RNU-44-RT \| 5'-GTTGGCTCTGGTGCAGGGTCCGAGGTATTCGCACC  AGAGCCAACAGTCAG-3' \| \| RNU-44-F \| 5'-GCCCTGGATGATGATAGCAA-3' \| \|  \|  \| \| ***for plasmid*** \| \| \| TMEM182-3'-UTR-WT-F \| 5'-CTCGAGTCACTAAATCAACTGTTGCC-3' \| \| TMEM182-3'-UTR-WT-R \| 5'-TCTAGACCAACAAAGCTCACATACCC-3' \| \| TMEM182-3'-UTR-DEL-F \| 5'-TTGAACATGTGCCTGATAATAGCTTTAATACCA TGACATGGGGAAAATCTCGATAG-3' \| \| TMEM182-3'-UTR-DEL-R \| 5'-ATTATCAGGCACATGTTCAATGAAAGGGAGACCTGT  TTACCTATTCCCTA-3' \| \| TMEM182-flag-F \| 5'-ATGAGACTAAATATCGCTATCTT-3’ \| \| TMEM182-flag-R \| 5'-CTT TGC GAC CTG CAT GAA CTC TATTACTTATCGTCGTCATCCTTGTAATCTCCCCCGTGATGTATCTGAATATGCC-3' \| \| TMEM182-GFP-F \| 5'-CCGCCTCGAGGCCACCATGAGACTAAATATCGCTATC  TT-3' \| \| TMEM182-GFP-R \| 5'-GCGGATCCCGGTGATGTATCTGAATATGCC-3' \| |
