# Supplementary material for: TNF-α-induced miR-450a mediates TMEM182 expression to promote oral squamous cell carcinoma motility
Source: PLoS One. 2019 Mar 20;14(3):e0213463. doi: 10.1371/journal.pone.0213463 (PMC6426234; doi:10.1371/journal.pone.0213463)
Supplement: S3 Table — (DOC) [file pone.0213463.s006.doc]

| **Table S3**. **Twelve of candidate genes downregulated by miR-450a.** | | | | | | | | |  |
| --- | --- | --- | --- | --- | --- | --- | --- | --- | --- |
| **Gene symbol*** | **Fold change**  **log_2_ ratio** | **Pearson *r* correlation ^§^** | | | | ***P* value ^#^** | | |  |
|  |
|  |  |  | | | |  | | |  |
| TMEM182 | 0.66 | -0.21551 | | | | 4.53E-08 | | |  |
| MIPOL1 | 0.65 | -0.1113 | | | | 6.36E-08 | | |  |
| KCNB1 | 0.49 | -0.06175 | | | | 1.11E-10 | | |  |
| PLN | 0.43 | -0.04892 | | | | 5.41E-15 | | |  |
| MYH11 | 0.5 | -0.01298 | | | | 6.81E-11 | | |  |
| UMODL1 | 0.6 | 0.000286 | | | | 1.97E-09 | | |  |
| FAM177B | 0.58 | 0.01673 | | | | 6.42E-08 | | |  |
| IRX6 | 0.25 | 0.040156 | | | | 1.67E-15 | | |  |
| GPR64 | 0.46 | 0.053378 | | | | 1.85E-09 | | |  |
| FAM149A | 0.34 | 0.056299 | | | | 3.33E-14 | | |  |
| GLB1L2 | 0.39 | 0.178384 | | | | 8.03E-11 | | |  |
| OGN | 0.23 | 0.315146 | | | | 3.15E-14 | | |  |
|  |  |  | | | |  | | |  |
| *Genes are listed in order of Pearson *r* correlation values from lowest to highest. | | | | | | | |  | |
| § Pearson *r* correlation between miR-450a and TMEM182 (log_2_ fold change). | | | | | | | |  | |
| # Student's t test, two-tails, two types. | | |  | |  | |  |  | |
|  | | | |  | | |  |  | |
